# Pituitary Adenylate Cyclase-Activating Polypeptide (PACAP) of the Bed Nucleus of the Stria Terminalis Mediates Heavy Alcohol Drinking in Mice

**DOI:** 10.1523/ENEURO.0424-23.2023

**Published:** 2023-12-15

**Authors:** Lauren Lepeak, Sophia Miracle, Antonio Ferragud, Mariel P. Seiglie, Samih Shafique, Zeynep Ozturk, Margaret A. Minnig, Gianna Medeiros, Pietro Cottone, Valentina Sabino

**Affiliations:** Laboratory of Addictive Disorders, Departments of Pharmacology and Psychiatry, Boston University Chobanian & Avedisian, School of Medicine, Boston, Massachusetts 02118

**Keywords:** addiction, alcohol, ethanol, anxiety, dependence, neuropeptide

## Abstract

Alcohol use disorder (AUD) is a complex psychiatric disease characterized by periods of heavy drinking and periods of withdrawal. Chronic exposure to ethanol causes profound neuroadaptations in the extended amygdala, which cause allostatic changes promoting excessive drinking. The bed nucleus of the stria terminalis (BNST), a brain region involved in both excessive drinking and anxiety-like behavior, shows particularly high levels of pituitary adenylate cyclase-activating polypeptide (PACAP), a key mediator of the stress response. Recently, a role for PACAP in withdrawal-induced alcohol drinking and anxiety-like behavior in alcohol-dependent rats has been proposed; whether the PACAP system of the BNST is also recruited in other models of alcohol addiction and whether it is of local or nonlocal origin is currently unknown. Here, we show that PACAP immunoreactivity is increased selectively in the BNST of C57BL/6J mice exposed to a chronic, intermittent access to ethanol. While pituitary adenylate cyclase-activating polypeptide (PACAP) type 1 receptor-expressing cells were unchanged by chronic alcohol, the levels of a peptide closely related to PACAP, the calcitonin gene-related neuropeptide, were found to also be increased in the BNST. Finally, using a retrograde chemogenetic approach in PACAP-ires-Cre mice, we found that the inhibition of PACAP neuronal afferents to the BNST reduced heavy ethanol drinking. Our data suggest that the PACAP system of the BNST is recruited by chronic, voluntary alcohol drinking in mice and that nonlocally originating PACAP projections to the BNST regulate heavy alcohol intake, indicating that this system may represent a promising target for novel AUD therapies.

## Significance Statement

Our results point at a key role for the neuropeptide PACAP (pituitary adenylate cyclase-activating polypeptide), specifically of the bed nucleus of the stria terminalis, in mediating heavy alcohol drinking in mice. This system may, therefore, represent a novel target for the treatment of alcohol use disorders.

## Introduction

Alcohol is the most common addictive substance in the world (see National Survey on Drug Use and Health, Table 5.4A—Alcohol Use Disorder in Past Year among Persons Aged 12 or Older, by Age Group and Demographic Characteristics: Numbers in Thousands, 2017 and 2018; available at: www.samhsa.gov). Every year, excessive alcohol use costs the United States $249 billion and causes 88,000 deaths, as well as various chronic diseases and social issues (Sacks et al., 2015). Alcohol use disorder (AUD), a highly prevalent, chronic, relapsing disorder, affects >14 million people in the United States alone, in addition to being severely undertreated, with only three modestly effective pharmacological therapies available (see National Survey on Drug Use and Health, Table 5.4A—Alcohol Use Disorder in Past Year among Persons Aged 12 or Older, by Age Group and Demographic Characteristics: Numbers in Thousands, 2017 and 2018; available at: www.samhsa.gov; Reus et al., 2018; Koob, 2021; Mason and Heyser, 2021).

Chronic exposure to ethanol has been shown to produce profound neuroadaptations in specific brain regions, including the recruitment of key stress neurotransmitters, ultimately causing allostatic changes that sustain excessive drinking (Koob, 2003, 2009; Breese et al., 2011). The bed nucleus of the stria terminalis (BNST) is a brain structure critically involved in the behavioral response to stress as well as in chronic, pathologic ethanol use (Kash et al., 2009, 2015; Pleil et al., 2015; Avery et al., 2016; Koob and Volkow, 2016; Lebow and Chen, 2016; Pati et al., 2020). Chronic ethanol causes hyperexcitability of and increased glutamatergic drive onto neurons of the laterodorsal BNST (BSTld), whose stimulation, in turn, results in an anxious phenotype, which may drive excessive drinking (Kash et al., 2009; Wills et al., 2012; Kim et al., 2013; Marcinkiewcz et al., 2015; Pleil et al., 2015; Marcinkiewcz et al., 2016).

The BSTld expresses an extraordinary amount of peptides, suggesting that diverse neuronal inputs may encode different signals allowing for fine-tuning of behavior (Kash et al., 2015). Pituitary adenylate cyclase-activating polypeptide (PACAP) is a 38 aa neuropeptide belonging to the secretin/glucagon/vasoactive intestinal polypeptide family, whose fibers, of nonlocal origin, are highly abundant in the BSTld (Ghatei et al., 1993; Piggins et al., 1996; Hannibal et al., 2002; Hammack et al., 2009; Missig et al., 2014; Stamatakis et al., 2014). PACAP is a key mediator of the stress response (Hashimoto et al., 2001; Agarwal et al., 2005; Stroth and Eiden, 2010; Ressler et al., 2011; Gaszner et al., 2012; Dore et al., 2013; Lehmann et al., 2013); PACAP administration elicits anxiety-like and anhedonic-like behavior (Dore et al., 2013; Missig et al., 2014; Lezak et al., 2014a; Hammack and May, 2015; Seiglie et al., 2015; Iemolo et al., 2016), and several types of stressors recruit the PACAP system, especially that of the BNST (Hannibal et al., 1995; Hammack et al., 2009; Stroth and Eiden, 2010; Kocho-Schellenberg et al., 2014; Missig et al., 2014; Lezak et al., 2014a; Seiglie et al., 2019; Varodayan et al., 2020; Seiglie et al., 2022).

Suggesting a likely involvement of the PACAP system in AUD, a single nucleotide polymorphism of the PACAP gene was found to be linked to high alcohol intake in a European population (Kovanen et al., 2010); furthermore, a specific genotype of the type I PACAP receptor (PAC1R) is associated with problematic alcohol use in women (Dragan et al., 2017). In the preclinical realm, the PACAP/PAC1R system has also been shown to be involved in the actions of alcohol by pharmacological and genetic deletion studies (Feany and Quinn, 1995; Tanaka et al., 2004, 2010; Gargiulo et al., 2020; Minnig et al., 2021, 2022). In particular, in the BNST, it has recently been shown that rats made dependent via chronic exposure to alcohol vapors display increased levels of PACAP, and that the pharmacological antagonism of PAC1R in this area is able to block dependence-induced excessive alcohol intake as well as anxiety-like behavior in these rats (Ferragud et al., 2021).

Whether the PACAP system of the BNST is also recruited in other models of alcohol addiction is currently unknown. Here, we first assessed PACAP immunoreactivity in the BNST of mice exposed for several weeks to chronic, intermittent access to ethanol. In the same model, we also measured the number of PAC1R-expressing cells and the levels of a peptide closely related to PACAP, the calcitonin gene-related neuropeptide (CGRP). We then used a retrograde approach to test the effect of chemogenetically inhibiting PACAP neuronal afferents to the BNST on ethanol consumption in the same model. Our data suggest that the PACAP system of the BNST is recruited by chronic, voluntary alcohol drinking in mice and that nonlocally originating PACAP projections to the BNST regulate heavy alcohol intake.

## Materials and Methods

### Subjects

Male and female C57BL/6J mice (7 weeks old on arrival) were purchased from The Jackson Laboratory. Group 1 was used for the time course and the PACAP immunohistochemistry (IHC; *n *=* *20 for time course; *n *=* *23 for the PACAP BNST IHC), group 2 was used for the PAC1R BNST IHC (*n *=* *26), and group 3 was used for CGRP BNST IHC (*n *=* *25). Male and female *Pacap-ires-Cre* mice, a gift from Bradford Lowell (Harvard Medical School, Boston, MA; Krashes et al., 2014; Ross et al., 2018) were bred in house onto a C57BL/6J background. Group 4 was used for the chemogenetic experiment (*n *=* *20). Mice were housed in an AAALAC-approved vivarium on a 12 h reverse light/dark cycle, with *ad libitum* access to water and regular rodent corn-based chow (Teklad Irradiated Global Rodent Diet 2918). Male and female mice were housed in the same vivarium room, though at a distance from each other. Experiments were conducted during the dark cycle of the animals. Procedures adhered to the National Institutes of Health *Guide for the Care and Use of Laboratory Animals* and *The Principles of Laboratory Animal Care* (eighth edition) and were approved by the Institutional Animal Care and Use Committee.

### Drugs

Ethanol solution (20% v/v) was obtained by diluting 200 proof ethanol in tap water. Clozapine-*N*-Oxide (CNO) was provided courtesy of Kenner Rice (National Institutes of Health) and was dissolved in sterile saline.

### Mouse intermittent access to two-bottle choice

Fifty milliliter conical tubes (Thermo Fisher Scientific) equipped with rubber stoppers and 2.5 inch straight metal-ball bearing sipper tubes (Ancare) were used for the delivery of solutions and were placed on top of the home cages. Mice were then given intermittent access using an intermittent access to two-bottle choice (IA2BC) paradigm for the entire duration of the experiments, during which time one water bottle was replaced with a bottle containing 20% (v/v) ethanol on alternating days for 24 h, as done previously (Hwa et al., 2011; Navarro et al., 2019; Quadir et al., 2020). Briefly, at the beginning of the dark cycle, preweighed bottles (one ethanol, one water) were provided, and 24 h later both bottles were removed and weighed again to calculate intake. Water controls received identical treatment, except that the bottles were filled with tap water. Additional cages and sets of bottles were used to ensure negligible spillage during cage handling. All groups of mice were exposed to the IA2BC paradigm.

#### Brain tissue preparation

Mice for IHC or fluorescence *in situ* hybridization (FISH) were deeply anesthetized and transcardially perfused with PBS followed by 4% paraformaldehyde (PFA). Brains were removed, postfixed at 4°C for 24 h, and then transferred to 30% sucrose at least until saturation. Brain sections were cut using a cryostat into 30 or 14 μm sections for IHC or FISH, respectively. For IHC, sections were stored in a cryoprotectant solution at −20°C until processed; for FISH, sections were mounted directly on slides, then dried at −20°C for 2 h, and finally stored at −80°C until the assay was performed.

#### Immunohistochemistry

##### PACAP IHC

Every fourth section (120 μm apart) of the BNST region (range, +0.38 to +0.02 mm from bregma) and every sixth section (180 μm apart) of the CeA region (range, −0.82 to −1.70 mm from bregma) were collected systematically and processed for IHC. Free-floating sections were washed in Tris-buffered saline (TBS) after every incubation. After rinsing, free-floating sections were incubated in a 0.3% hydrogen peroxide TBS solution to quench endogenous peroxidases, followed by additional rinsing and a blocking step (3% normal goat serum in 0.4% Triton X-100). Sections were then incubated in anti-PACAP primary antibody [1:1000; catalog #T-4473, Bachem (RRID: AB_519166); Piggins et al., 1996; Norrholm et al., 2005; Das et al., 2007; Csati et al., 2012; Nakamura et al., 2014; Matsumoto et al., 2016; Steinberg et al., 2016; Liu and Wong-Riley, 2019; Meloni et al., 2019] in blocking solution for 24 h at 4°C. Sections were then incubated in a biotinylated anti-rabbit secondary antibody (1:500; Vector Laboratories) in blocking solution for 2 h at room temperature (RT). Sections were then incubated in secondary antibody (1:500; biotinylated anti-rabbit, Vector Laboratories) in blocking solution for 2 h at room temperature, and, finally, incubated in an avidin–biotin horseradish peroxidase ABC complex solution (Vector Laboratories) in blocking solution for 1 h. Sections were then processed using a diaminobenzidine (DAB) substrate kit (Vector Laboratories) until reaction was complete, mounted onto slides, and allowed to dry overnight. The following day, slides were dehydrated and coverslipped using DPX Mountant (Electron Microscopy Sciences).

##### PAC1R and CGRP IHC

Every fourth section (120 μm apart) of the BNST region (range, +0.38 to +0.02 mm from bregma) was processed. Free-floating sections were washed in TBS after every incubation. Following initial TBS washes, 10 mm sodium citrate buffer, pH 6, antigen retrieval was performed at 80°C for 30 min. Sections were blocked with a blocking solution (3% normal goat serum in 0.2% Triton X-100 in TBS) for 1 h at RT, followed by incubation with primary antibody [1:250, for 48 h at 4°C; anti-PAC1R; catalog #AVR-003 Alomone Labs (RRID: AB_2756805); 1:250, for 24 h at 4°C; anti-CGRP; catalog #ab81887, Abcam (RRID: AB_1658411)]. Sections were then incubated with secondary antibody (for PAC1R: 1:200; anti-rabbit; catalog #AF555, Thermo Fisher Scientific; for CGRP: anti-mouse; 1:250; catalog #AF488, Thermo Fisher Scientific). Sections were then washed in TBS and coverslipped with DAPI-containing mounting medium (VECTASHIELD Antifade Mounting Medium with DAPI, Vector Laboratories).

#### Quantification

##### PACAP density quantification

To assess PACAP immunoreactivity, chromogen PACAP pictures were taken in bright field at a 10× magnification under a preset exposure and gain to standardize the images. For each image, area contours were drawn corresponding to the laterodorsal subdivision (oval nucleus) of the BSTld or the capsular subdivision (CeC) and lateral subdivision (CeL) of the central nucleus of the amygdala (CeA) along the entire bregma range, with a microscope (model BX-51, Olympus) equipped with a live video camera (model Retiga 2000R, QImaging) and a three-axis motorized stage (model MAC6000 XYZ, Ludl Electronics). Densitometry analysis was then performed using the ImageJ software (NIH), where images were converted to 8 bit and adjusted using the autothreshold Triangle algorithm. Once converted, the mean optical density of signal was calculated by subtracting the background signal and then by normalizing the value to the traced area. The final number of brains was *n *=* *23 for PACAP BNST and *n *=* *29 for PACAP CeA IHC from *n *=* *32 (only brains with good staining and tissue quality were used).

##### CGRP density quantification

To assess CGRP immunoreactivity, fluorescent pictures of sections containing the BSTld were captured as described above, while imaging parameters were held constant for all images. Three digital squares were placed randomly within the staining region, and the mean labeling intensity of the three squares was determined. The final number of brains was *n *=* *26 for CGRP BNST IHC.

##### PAC1R-positive cell quantification

The number of PAC1R-expressing cells was quantified using an unbiased stereological approach as previously described (Ferragud et al., 2020, 2021; Minnig et al., 2021). The BSTld was outlined with an Olympus PlanApo N 2× objective (numerical aperture, 0.08), and counting was done using an Olympus UPlanFL N 20× objective (numerical aperture, 0.75). Counts were performed by an experimenter blind to conditions using a grid and a counting frame of 250 × 250 μm, a guard zone of 2 μm, and a dissector height of 20 μm in the Optical Fractionator Workflow module in Stereo Investigator. The final number of brains was *n *=* *25 for PAC1R BNST IHC.

#### Stereotaxic surgery

*Pacap-ires-Cre* mice were 7–10 weeks of age at the time of surgery. Surgeries were performed using a Kopf stereotaxic frame, using isoflurane for anesthesia. BNST coordinates were as follows: anteroposterior, +0.85; mediolateral, ±0.90; dorsoventral, –4.10 (from skull). A retrograde adeno-associated virus (AAV) expressing a Cre-dependent inhibitory designer receptor exclusively activated by designer drugs (DREADD), AAVrg-hSyn-DIO-hM4D(Gi)-mCherry, was delivered bilaterally into the BNST (0.3 μl/side) via a 22 gauge syringe (2 μl neurosyringe, Hamilton) over the course of 5 min, with an additional 5 min wait to avoid backflow. Approximately 1 week after surgery, the IA2BC paradigm began, and drug testing began ∼7 weeks after surgery (after 6 weeks of ethanol drinking). At the end of testing (∼13 weeks after surgery), mice were killed by isoflurane anesthesia, and brains were collected after transcardial perfusion for brain histology.

#### Clozapine-*N*-oxide administration

For DREADD experiments, CNO was administered intraperitoneally (0, 1, and 3 mg/kg, in a volume of 10 ml/kg in saline) 30 min before the beginning of the drinking session, using a within-subject Latin-square design. Mice were allowed at least one treatment-free drinking session between test days. CNO is a pharmacologically inert metabolite of the atypical antipsychotic clozapine; although there is a slight risk of CNO back-metabolizing to clozapine, studies have shown that it has little or no pharmacological activity in mice and rats when administered at the recommended doses (Urban and Roth, 2015), and previous studies have shown that CNO itself does not impact alcohol intake (den Hartog et al., 2016; Kreifeldt et al., 2022; Flanigan et al., 2023; Griffin et al., 2023; Zamudio et al., 2023). Alcohol, water, and food weights were recorded at 2, 6, and 24 h, common time points used in the literature (Sabino et al., 2013; Newman et al., 2018; Quadir et al., 2021a, b).

#### Histology in DREADD mice

Mice were deeply anesthetized with isoflurane and transcardially perfused with ice-cold heparinized PBS, followed by 4% paraformaldehyde. Brains were extracted, allowed to fix in 4% paraformaldehyde for 24 h, and then placed in 30% sucrose for cryoprotection. Brains were sliced at 30 μm on a cryostat and then stored at −20°C in cryoprotectant. Sections containing the BNST were mounted on slides and coverslipped with VECTASHIELD Antifade Mounting Media, and the injection tract was verified under a microscope. A few slices containing the lateral parabrachial nucleus (LPB) were also collected to verify successful retrograde transport of the virus (mCherry expression) in an upstream area as well as to verify the specificity of the virus for PACAP neurons (see next paragraph).

#### Fluorescence *in situ* hybridization

RNAscope Multiplex Fluorescent Reagent Kit version 2 (ACD) was used following the manufacturer instructions. Slides were first baked at 60°C for 30 min, fixed in 4% PFA, and dehydrated in ethanol. Sections were then incubated with hydrogen peroxide (10 min, RT), then boiled in target retrieval solution for 5 min and surrounded by a hydrophobic barrier (ImmEdge Pen, Vector Laboratories). Sections were incubated in Protease Plus (30 min, RT), followed by incubation with target probes (Adcyap1, catalog #405911; Tac1, catalog #410351; mCherry, catalog #431201; all from ACD) for 2 h at 4°C. Signal was then amplified using amplifiers AMP1-3. Following amplification, each probe was assigned a TSA Plus fluorophore (FITC, Cy3, Cy5; for 30 min at 40°C) and corresponding channel (C1, C2 and C3, 15 min at 40°C), each channel step concluding with an HRP blocker. Sections were incubated in DAPI for 30 s at RT and then coverslipped in mounting medium (Vectashield Hardset AntiFade Mounting Medium, Vector Laboratories). Slides were imaged using a VS120 Virtual Slide Scanner (Olympus) at a 20× magnification. Images were then processed using QuPath version 0.4.3 software.

### Statistical analysis

Data from the IHC studies were analyzed using either two-way ANOVAs (Sex and Group as between-subjects factors) or with Student’s *t* tests when sexes were pooled. Data from the alcohol-drinking study were analyzed using two-way ANOVAs (Dose of CNO and Time as within-subject factors). Pairwise *post hoc* comparisons were made using the Student–Newman–Keuls test or a Student’s *t* test when comparing two groups. Significance was set at *p *<* *0.05. The software/graphic packages used were StatSoft Statistica 12 and GraphPad Prism 8.

## Results

### Acquisition of drinking in males and female C57BL/6J mice in IA2BC paradigm

**Figure 1.**
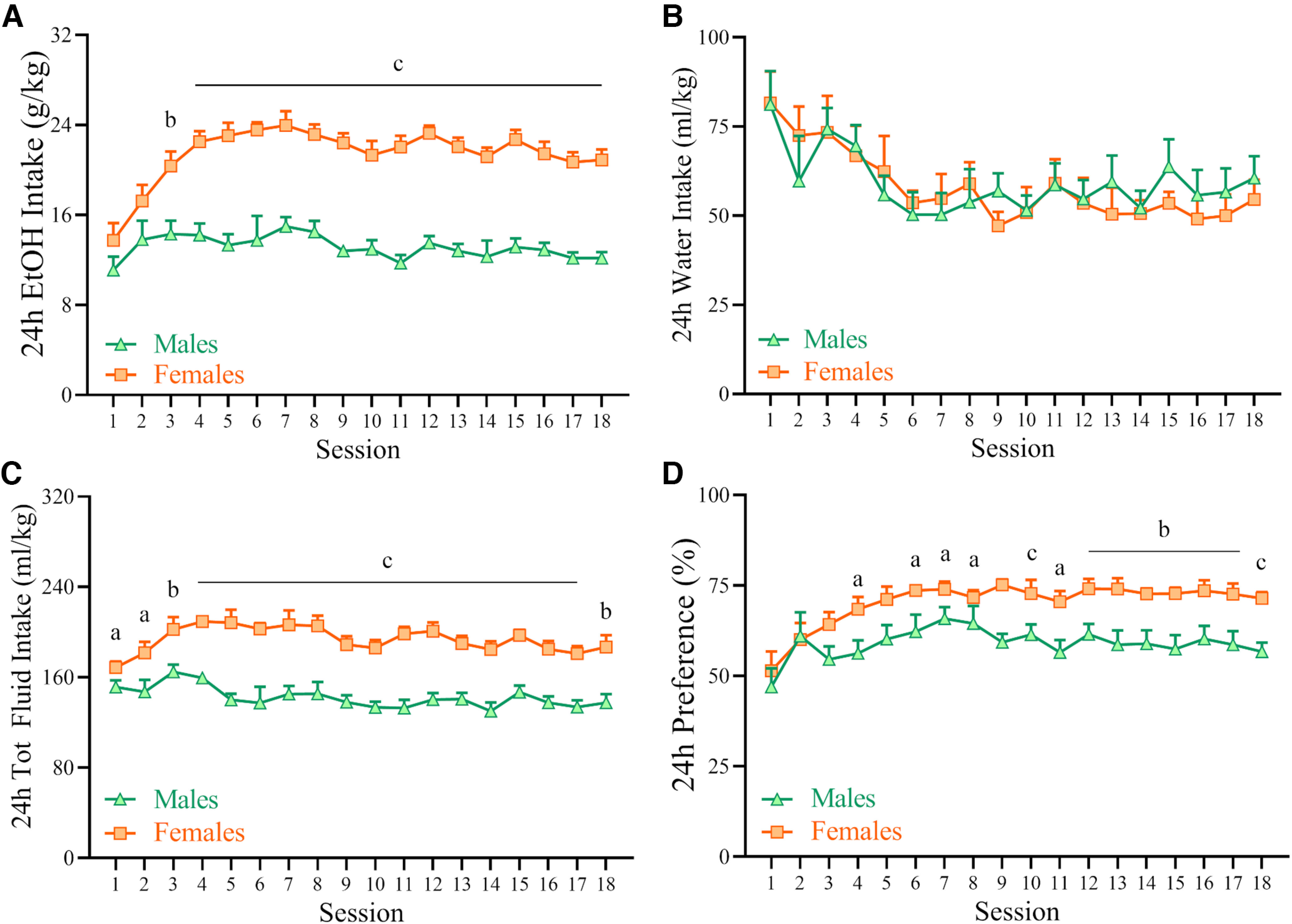
Male and female C57BL/6 mice were exposed to intermittent access to alcohol (IA2BC; Monday, Wednesday, Friday, 24 h/d, 20% v/v ethanol vs water) for 6 consecutive weeks or 18 drinking sessions. ***A***, ***C***, ***D***, Female mice drank more alcohol than males (***A***), drank more fluids (***C***), and showed a higher preference for alcohol (***D***). ***B***, Water intake did not significantly differ between sexes. Data represent the mean ± SEM (*n *=* *10/group, total 20 mice). a, *p* < 0.05; b, *p* < 0.01; c, *p* < 0.001; versus males.

C57BL/6J mice given intermittent access to 20% v/v ethanol and water (IA2BC) increased their intake of alcohol across the time of observation (6 weeks), as shown in Figure 1 (Session: *F*_(17,306)_ = 5.59, *p *<* *0.001). Female mice showed significantly higher ethanol intake compared with male mice (Sex: *F*_(1,18)_ = 124.51, *p *<* *0.001; Sex * Session: *F*_(17,306)_ = 2.57, *p *<* *0.001), as shown in Figure 1*A*. Female mice rapidly increased their ethanol drinking behavior (escalation), with a significant difference between sexes evident already on day 3. The average ethanol intake in session 18 was 20.9 and 12.2 g/kg in females and males, respectively. Intake of water decreased across time (Session: (*F*_(17,306)_ = 4.10, *p *<* *0.001) consistently in both sexes [Sex: *F*_(1,18)_ = 0.077; not significant (n.s.); Sex * Session: *F*_(17,306)_ = 0.51, n.s.; Fig. 1*B*]; the average water intake in session 18 was 54.5 and 60.5 ml/kg in females and males, respectively. Total fluid intake was also higher in female mice (Sex: *F*_(1,18)_ = 77.78, *p *<* *0.001; Sex * Session: *F*_(17,306)_ = 1.63, *p *=* *0.055), as shown in Figure 1*C*; the average total fluid intake in session 18 was 186.7 and 137.5 ml/kg in females and males, respectively. Ethanol preference was also higher in female mice throughout the observation period (Sex: *F*_(1,18)_ = 17.02, *p *<* *0.001; Sex * Session: *F*_(17,306)_ = 1.05, n.s.); the average preference for ethanol in session 18 was 71.4% and 56.7% in females and males, respectively.

### Chronic, intermittent ethanol drinking increases PACAP levels in the BNST

**Figure 2.**
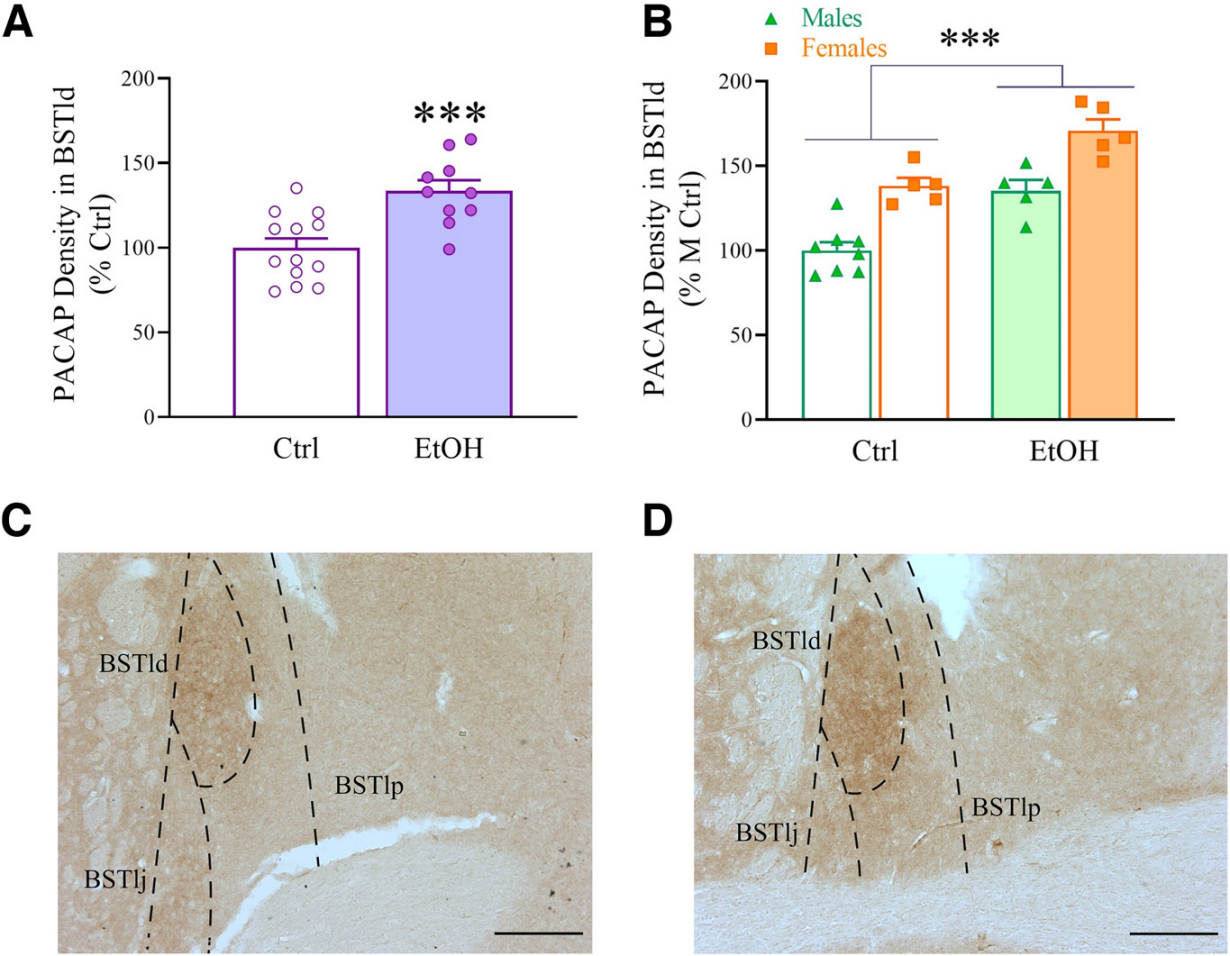
***A***, PACAP immunoreactivity levels are elevated in the BSTld of male and female C57BL/6J mice exposed to intermittent access to alcohol (IA2BC; EtOH), compared with control, water-only mice (Ctrl). ***B***, Data are reported with the two sexes pooled. ***C***, ***D***, Representative PACAP staining (DAB) in mouse BNST of a Ctrl and an EtOH subject, respectively. Magnification, 10×. Scale bar, 200 μm. Mice were killed after 7 weeks of IA2BC, 24 h after the end of the last drinking session. BSTlp, BNST lateral-posterior; BSTlj, BNST lateral-justacapsular. Data represent the mean ± SEM (***A***, *n *=* *10–13/group; ***B***, *n *=* *5–8/group; total, 23 mice). ***p < 0.001 versus Ctrl. Extended Data Figure 2-1 shows the effect of IA2BC on PACAP immunoreactivity in the CeA.

After 7 weeks of IA2BC access, IA2BC and control mice were killed 24 h after the last drinking session. Brain slices were processed for PACAP IHC and DAB staining quantified by densitometry. Average ethanol intake during the last session in these animals was as follows: males, 12.1 ± 1.0 g/kg; females, 20.3 ± 1.5 g/kg. As shown in Figure 2*A*, mice with a history of chronic, intermittent alcohol drinking displayed higher levels of PACAP in the BNST in both male and female mice (Ethanol: *F*_(1,19)_ = 34.38, *p *<* *0.001; Ethanol * Sex: *F*_(1,19)_ = 0.06, n.s.). A main effect of Sex was also observed, with female mice showing higher levels of PACAP in this region (Sex: *F*_(1,19)_ = 40.13, *p *<* *0.001). Figure 2*B* shows the data not dissociated by sex; IA2BC overall caused a 33.5% increase in PACAP levels in the BSTld. As previously shown, across the anterior–posterior axis of the BNST, the majority of PACAP immunoreactivity was found in the BSTld (Missig et al., 2014; Seiglie et al., 2019; Jiang et al., 2023), and the PACAP immunohistochemical staining pattern was found to be limited to incoming fibers, with no local cell bodies labeled. On the other hand, no statistically significant increase in PACAP immunoreactivity was observed in the CeA (capsular and lateral subdivisions) of IA2BC mice compared with controls, as shown in Extended Data Figure 2-1, although a trend to an Ethanol * Sex interaction could be observed (*p *=* *0.08).

10.1523/ENEURO.0424-23.2023.f2-1 Figure 2-1 ***A***, PACAP levels are unaltered in the CeA of male and female C57BL/6J mice exposed to intermittent access to alcohol (IA2BC; EtOH), compared with control, water-only mice (Ctrl). ***B***, Data are reported with the two sexes pooled. ***C***, ***D***, Representative PACAP staining (DAB) in mouse BNST of a Ctrl and an EtOH subject, respectively. Magnification, 10×. Scale bar, 200 μm. Mice were killed after 7 weeks of IA2BC, 24 h after the end of the last drinking session. CeM, CeA medial. Data represent the mean ± SEM (***A***, *n *=* *14–15/group; ***B***, *n *=* *6–8/group). Download Figure 2-1, TIF file.

### Chronic, intermittent ethanol drinking does not affect the number of PAC1R-positive cells in the BNST

**Figure 3.**
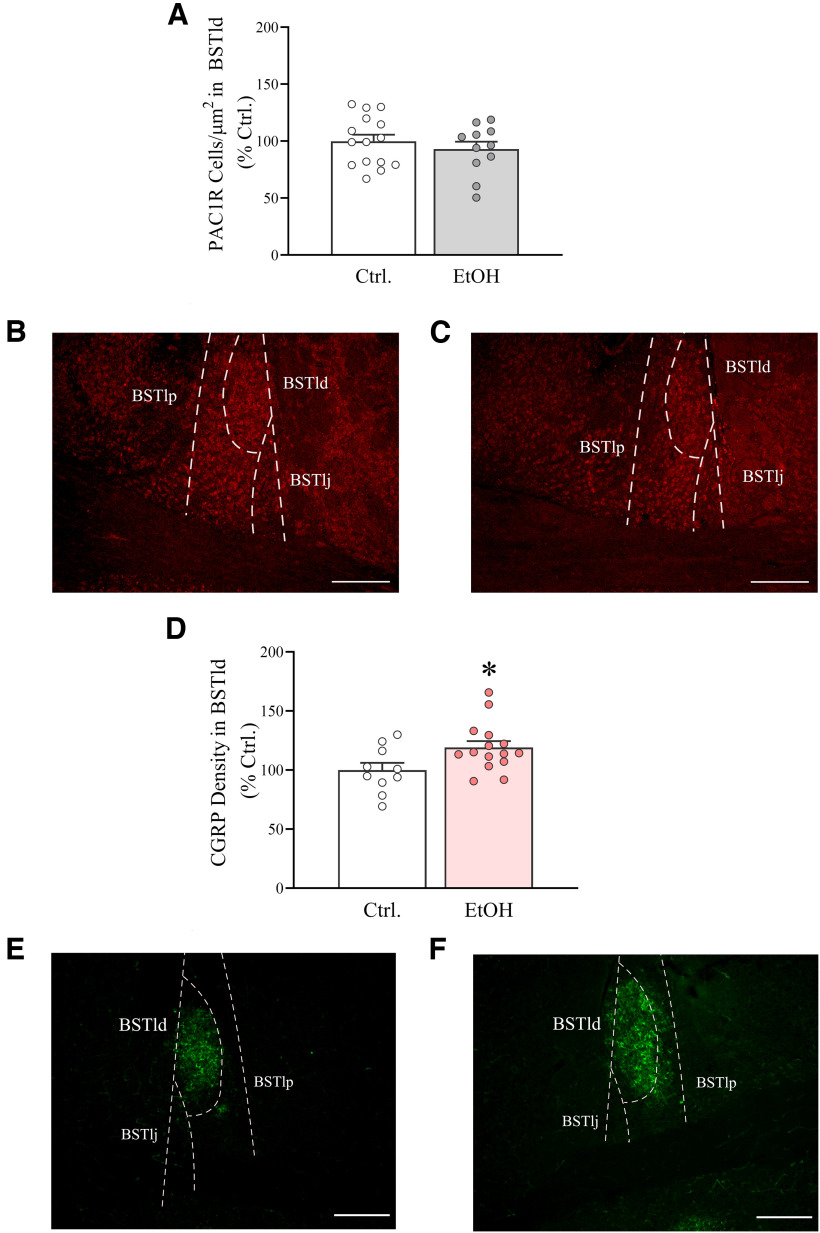
***A***, The number of PAC1R-immunoreactive cells is not altered in the BNST of IA2BC mice. ***B***, ***C***, Representative PAC1R staining (fluorescence) in mouse BNST of a Ctrl and an EtOH subject, respectively. Magnification, 10×. Scale bar, 200 μm. ***D***, CGRP levels are elevated in the BSTld of IA2BC mice (EtOH), compared with control mice (Ctrl). ***E***, ***F***, Representative CGRP staining (fluorescence) in mouse BNST of a Ctrl and an EtOH subject, respectively. Magnification, 10×. Scale bar, 200 μm. Mice were killed 24 h after the end of the last IA2BC session. BSTlp, BNST lateral-posterior; BSTlj, BNST lateral-justacapsular. Data represent the mean ± SEM (***A***, *n *=* *11–15/group; ***D***, *n *=* *10–15/group; total, 26 mice). *p < 0.05 versus Ctrl.

IA2BC and control mice were killed 24 h after the last drinking session. Brain slices were processed for PAC1 IHC, and immunofluorescent staining was quantified by cell counting. Average ethanol intake during the last session in these animals was as follows: males, 15.2 ± 1.4 g/kg; females, 21.5 ± 1.2 g/kg. No difference in the number of PAC1R-positive cells was found in the BSTld between mice with a history of chronic, intermittent ethanol drinking and control mice in either sex (Ethanol: *F*_(1,22)_ = 0.53, n.s.; Ethanol * Sex: *F*_(1,22)_ = 0.003, n.s.). Figure 3*A* shows the data after pooling the two sexes. PAC1R immunoreactivity was found to be present also in the other subdivisions of the dorsal BNST, where similarly no group differences were found (data not shown).

### Chronic, intermittent ethanol drinking increases CGRP levels in the BNST

Since PACAP immunoreactivity significantly overlaps with that of CGRP in the fibers of extended amygdala (Missig et al., 2014), we assessed whether IA2BC mice also displayed increased CGRP levels in the BNST. Average ethanol intake during the last session in these animals was as follows: males, 13.6 ± 1.3 g/kg; females, 20.7 ± 2.1 g/kg. We observed that BNST CGRP expression was highest within the BSTld, and that its localization closely mimics PACAP expression in this area, as previously shown. IA2BC mice displayed higher levels of CGRP in the BSTld in both sexes (Ethanol: *F*_(1,21)_ = 4.65, *p *<* *0.05; Ethanol * Sex: *F*_(1,21)_ = 0.05, n.s.). Interestingly, unlike with PACAP, no sex differences in CGRP levels could be observed in this region (Sex: *F*_(1,21)_ = 0.20, n.s.). Figure 3*D* shows the data after pooling the two sexes; IA2BC overall caused a 19.2% increase in CGRP levels.

### Inhibition of afferent PACAP projections to the BNST reduced heavy ethanol drinking in mice

**Figure 4.**
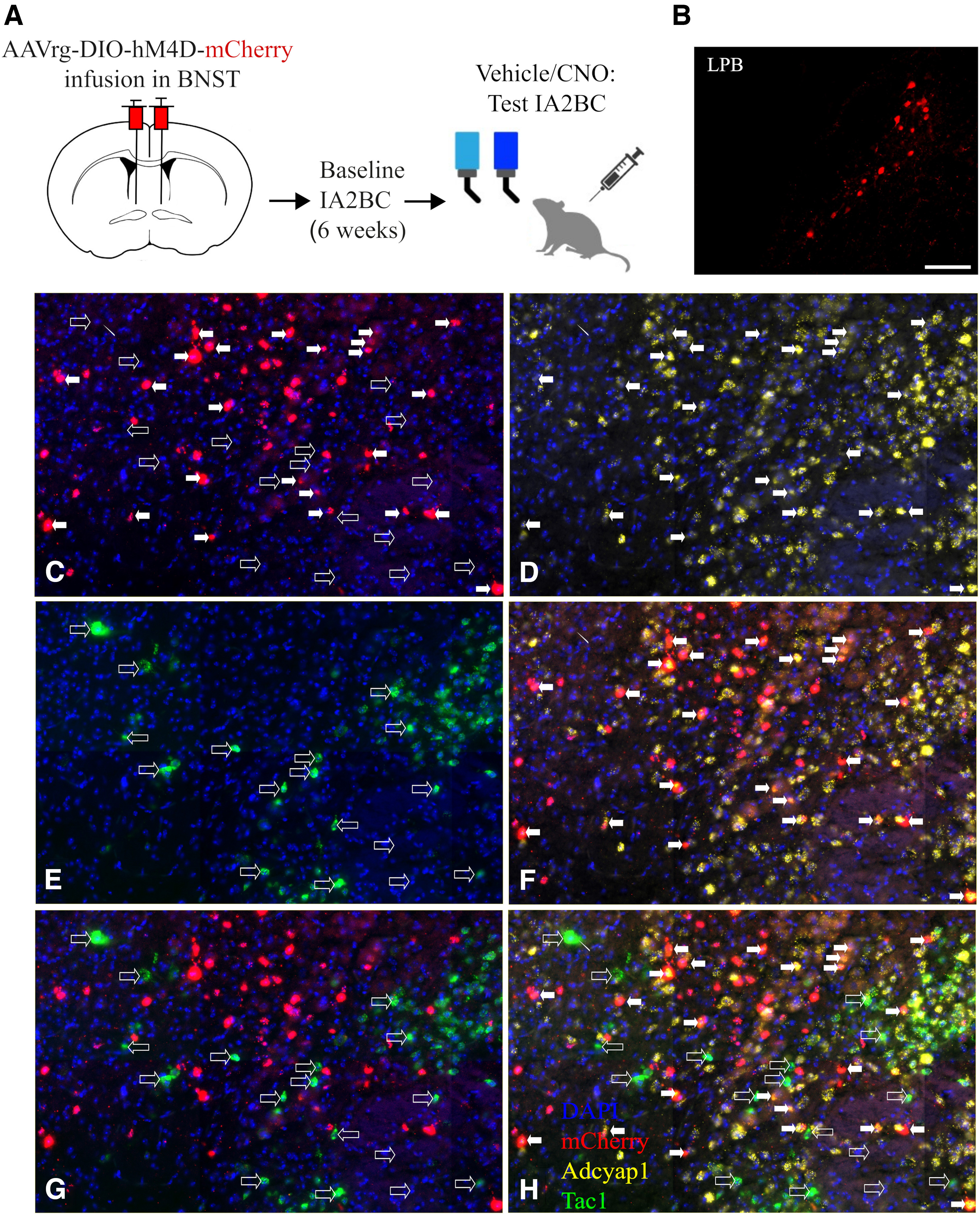
***A***, Scheme and timeline of the chemogenetic experiment. ***B***, Representative picture of mCherry fluorescence in the LPB. Scale bar, 100 μm. ***C–H***, Verification (by *in situ* hybridization) of the selectivity of the retrograde AAV [AAVrg-hSyn-DIO-hM4D(Gi)-mCherry] for Adcyap1, and not Tac1, neurons in the LPB. Filled arrows indicate representative mCherry-expressing cells (where coexpression with Adcyap1 is visible); empty arrows indicate representative Tac1 (where absence of mCherry is visible). Extended Data Figure 4-1 shows the location of the AAVrg-hSyn-DIO-hM4D-mCherry infusions in the BNST.

As shown in the scheme in Figure 4*A*, a group of *Pacap-ires-Cre* mice were infused with a Cre-dependent retrograde virus expressing an hM4Di inhibitory DREADD expressing the fluorophore mCherry in the BNST. Extended Data Figure 4-1 shows the viral injection sites in the BNST; 7 of the 20 mice were removed because of absent or inaccurate injection placement (*n *=* *13). As expected, mCherry was detected upstream in the LPB, as shown in Figure 4*B*. Importantly, as assessed with fluorescence *in situ* hybridization, the Cre dependency of the virus was verified as the expression of mCherry was restricted to PACAP-expressing cells. Indeed, mCherry was colocalized with PACAP (Adcyap1 gene, >95%) but not with tachykinin (Tac1 gene), another neuropeptide widely expressed in LPB, as shown in Figure 4*C–G*. The totality of PACAP-expressing neurons expressed mCherry only partially, consistent with the Cre line being heterozygous for Cre and with the spatial limitations of the viral spread. In addition, some neurons appeared to express both Adcyap1 and Tac1, in line with previous literature.

10.1523/ENEURO.0424-23.2023.f4-1 Figure 4-1 ***A***, Illustrations of coronal rat brain slices; numbers represent the distance from bregma (mm). Symbols represent the injection sites of the AAVrg-hSyn-DIO-hM4D-mCherry in the BNST (each dot is the location of one injection, each star the location of 2 or more injections). Download Figure 4-1, TIF file.

**Figure 5.**
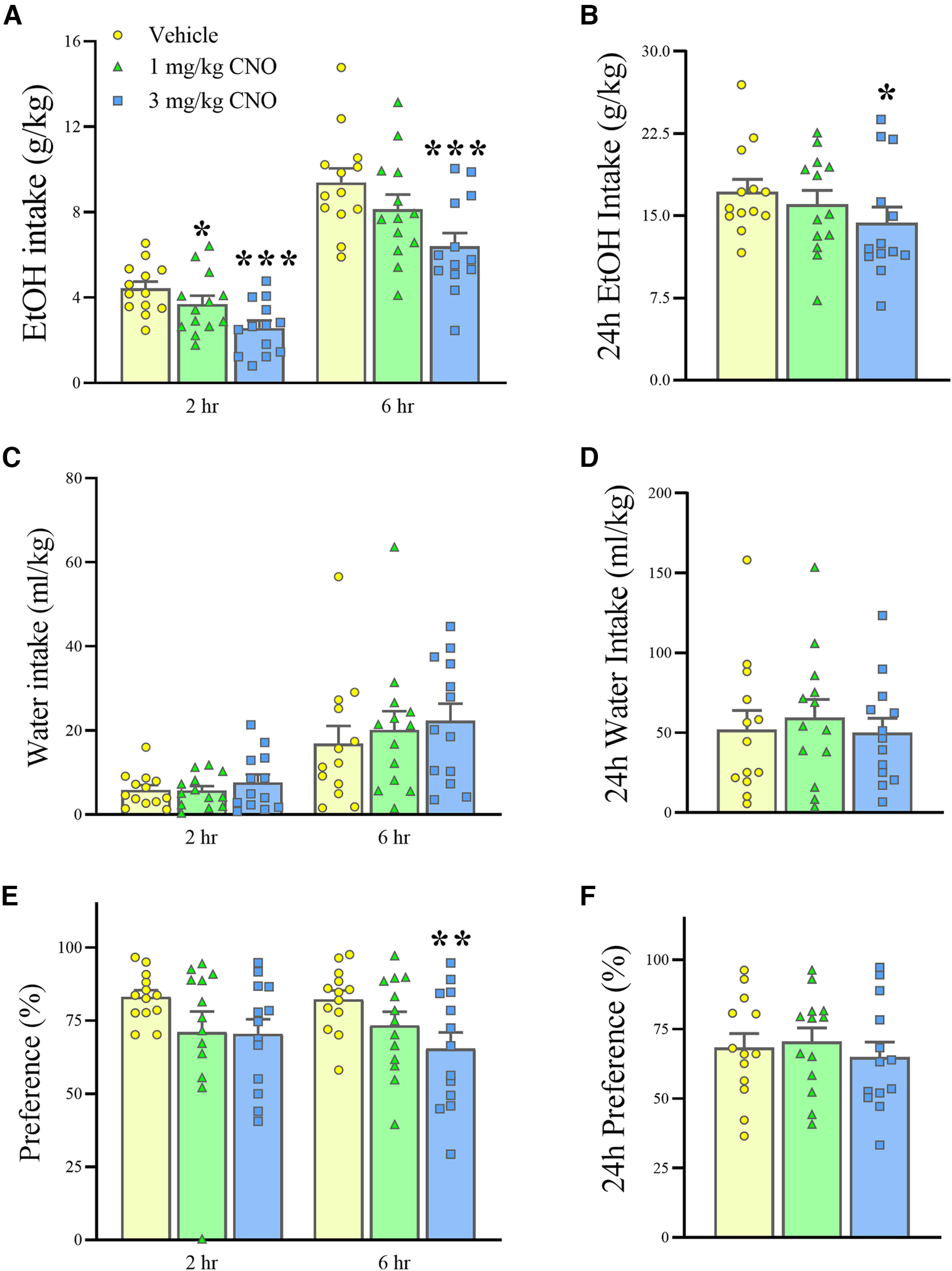
***A–F***, The effect of inhibition of PACAP neurons afferent to the BNST on ethanol intake (***A***, ***B***), water intake (***C***, ***D***), and ethanol preference (***E***, ***F***). Administration of CNO (0–3 mg/kg, i.p.) dose-dependently reduced 2, 6, and 24 h ethanol intake in AAVrg-hSyn-DIO-hM4D(Gi)-mCherry BNST-infused mice, while it did not affect water intake. CNO also reduced 6 h ethanol preference. Data represent the mean ± SEM (*n *=* *13 mice). *p < 0.05, **p < 0.01, ***p < 0.001; versus Vehicle.

In AAVrg-hSyn-DIO-hM4D(Gi)-mCherry-infused mice exposed to chronic, intermittent ethanol access, administration of the selective DREADD ligand CNO (0–3 mg/kg, i.p.) dose-dependently reduced the incremental ethanol intake at both the 2 and 6 h time points, in both sexes (Dose: *F*_(2,22)_ = 11.01, *p *<* *0.001; Dose * Time: *F*_(2,22)_ = 0.83, n.s.; Dose * Sex: *F*_(2,22)_ = 1.58, n.s.). *Post hoc* analyses revealed that, at the 2 h time point, both doses of CNO (1 and 3 mg/kg) significantly reduced ethanol intake (−16.6% and −41.8%, respectively) as shown in Figure 5*A* (male and female data pooled); at the 6 h time point, only the 3 mg/kg dose significantly decreased intake (−31.8%). The effect of CNO persisted when looking at the cumulative 24 h ethanol intake (Dose: *F*_(2,24)_ = 4.04, *p *<* *0.05), with the highest dose causing an overall 16.6% reduction (Fig. 5*B*). However, in line with the reported CNO half-life, no effect of CNO could be observed on the incremental 6–24 h ethanol intake (Dose: *F*_(2,24)_ = 0.05, n.s.).

Water intake was not significantly affected by the CNO treatment in either sex either at the 2 and 6 h time points (Dose: *F*_(2,22)_ = 0.60, n.s.; Dose * Time: *F*_(2,22)_ = 0.31, n.s.; Dose * Sex: *F*_(2,22)_ = 1.19, n.s.) or at the 24 h time point (Dose: *F*_(2,24)_ = 0.44, n.s.), as shown in Figure 5, *C* and *D*.

Preference for the ethanol solution showed a very strong trend to be affected by CNO treatment (Dose: *F*_(2,22)_ = 2.40, *p* = 0.11; Dose * Time * Sex: *F*_(2,22)_ = 3.44, *p *=* *0.050), with a significant effect observed at the 6 h time point by the 3 mg/kg dose, as revealed by *post hoc* analysis (−20.5%), as shown in Figure 5*E*. No significant effect on ethanol preference persisted at 24 h (Fig. 5*F*).

Finally, food intake was not significantly affected by the CNO treatment in either sex at either the 2 and 6 h time points (Dose: *F*_(2,22)_ = 1.00, n.s.; Dose * Time: *F*_(2,22)_ = 0.02, n.s.; Dose * Sex: *F*_(2,22)_ = 1.11, n.s.) or at the 24 h time point (Dose: *F*_(2,24)_ = 0.07, n.s.; data not shown).

## Discussion

The main findings of this series of studies were as follows: (1) chronic, intermittent ethanol drinking increases PACAP levels in the BNST, but not the CeA, of mice; (2) chronic, intermittent ethanol drinking does not affect the number of PAC1R-expressing cells in the BNST; (3) chronic, intermittent ethanol drinking also increases CGRP levels in the BNST; and (4) inhibition of afferent PACAP projections to the BNST is sufficient to reduce heavy ethanol drinking in mice.

Our immunohistochemistry data revealed an increased expression of PACAP-positive fibers specifically within the BSTld of mice exposed to 7 weeks of chronic, intermittent ethanol drinking, compared with control, water-drinking mice (+33.5%). This increase was observed 24 h after the end of the last drinking session, hence during acute withdrawal, when alcohol was no longer onboard. Our results in this mouse model of heavy alcohol drinking are consistent with previous studies that have shown increased PACAP expression in the BSTld in rats following chronic passive exposure to ethanol vapors (Ferragud et al., 2021), following chronic cocaine administration (Miles et al., 2018), and also following chronic variable stress (Hammack et al., 2009), suggesting that PACAP is recruited specifically in the BNST following chronic exposure to both stressors or drug and alcohol exposure. PACAP immunoreactivity in the BSTld, as well as the CeA (CeC and CeL subdivisions), appears as fibers, consistently with previous results from our and other laboratories and strengthening the notion that PACAP is not locally produced, but rather that these fibers are afferent projections from other brain areas (Piggins et al., 1996; Hannibal, 2002; Missig et al., 2014; Seiglie et al., 2019). Interestingly, in our mouse IA2BC model, PACAP levels were not significantly altered in the CeA, a brain region also part of the extended amygdala, which receives similar afferent projections, suggesting a selective effect in the BNST, in line with our previous finding in the rat dependence model (Ferragud et al., 2021). Notably, the number of PAC1R-positive cells, as assessed by immunohistochemistry, did not differ in the BSTld of ethanol drinking mice compared with controls; future studies incorporating different stages of ethanol exposure and withdrawal could, however, provide more comprehensive insights into the potential impact of chronic ethanol exposure on the expression of PAC1R-positive cells.

We also observed a significant increase in the levels of the neuropeptide CGRP in the BSTld of ethanol drinking mice, compared with controls (+19.2%). CGRP is a 37 aa peptide whose immunoreactivity displays considerable overlap with PACAP in the BNST, both being particularly abundant in fibers of the BSTld (Dobolyi et al., 2005; Hammack et al., 2009; Sink et al., 2011). It was shown that the majority of PACAP-expressing fibers in this region appears to coexpress CGRP (Missig et al., 2014), suggesting that these two neuropeptides may be coreleased from the same neurons, which likely originate in the LPB (Missig et al., 2014; Zhang et al., 2021; Jiang et al., 2023; Seiglie et al., 2023). CGRP also shares similarities with PACAP in terms of functions; CGRP has been shown to play an important role in the regulation of stress and anxiety-like behaviors, physical and emotional pain responses, migraine, and taste aversion (Kocorowski and Helmstetter, 2001; Campos et al., 2018; Chen et al., 2018). Despite the similarities, however, some recent studies in the context of migraine have suggested that the two peptides may act by independent mechanisms, possibly by distinct intracellular signaling pathways (Ernstsen et al., 2022; Kuburas and Russo, 2023). Interestingly, while an anxiogenic-like and pronociceptive role of CGRP in the CeA has been described (Han et al., 2005, 2010), much less is known about the function of CGRP in the BNST, warranting the need for further studies. The role of CGRP in alcohol addiction is still unclear. Alcohol directly evokes the release of CGRP in the trigeminal ganglia (Nicoletti et al., 2008). Early studies have also found lower basal CGRP levels in multiple brain regions of Indiana P alcohol-preferring rats, at the same time as increased levels during protracted withdrawal after ethanol vapor administration (Hwang et al., 1995; Ehlers et al., 1999). More recently, intermittent alcohol drinking during adolescence was shown to increase CGRP levels in the brainstem of rats (Tringali et al., 2023). In addition, in line with our current findings, continuous access to alcohol drinking was shown to increase CGRP levels in the BNST of Sardinian alcohol-preferring rats (Rossetti et al., 2019). The finding of increased levels of CGRP after heavy alcohol drinking in the BNST of C57BL/6 mice is, therefore, an interesting one, and the potential functional relevance of this change for alcohol drinking and associated negative states warrants further investigation.

The use of an AAV produced with a retrograde serotype permits retrograde access to projection neurons. Specifically, to assess the significance of the increased PACAP levels in the BNST following intermittent alcohol drinking, we used a retrograde chemogenetic approach in *Pacap-ires-Cre* mice to Cre-dependently inhibit PACAP neurons that project to the BNST and then measured ethanol intake. Our results show that the chemogenetic inhibition of PACAP afferents to the BNST is sufficient to reduce ethanol drinking behavior. The effect was more marked at the 2 h time point, with both doses of CNO producing a significant reduction in ethanol intake (−16.6% and −41.8%, compared with vehicle treatment, respectively), consistent with the short half-life of the DREADD ligand CNO. The effect of the high dose of CNO, however, was still evident at the 6 h (−31.8%) and 24 h (−16.6%) time points, and ethanol preference was also significantly reduced at the 24 h time point. Water intake was not affected by the PACAP pathways inhibition, ruling out the alternative explanation that the manipulation may have induced malaise.

The present data suggest that neuronal pathways that express PACAP and project to the BNST contribute to the high levels of alcohol drinking seen in this mouse model. Stressors have been shown to increase PACAP levels within the BNST and intra-BNST PAC1R antagonism attenuates the response to stress as well as stress-induced reinstatement of cocaine-seeking behavior (Roman et al., 2014; Lezak et al., 2014b; Miles et al., 2018; Seiglie et al., 2019, 2022). Recently, it has been shown that antagonism of PAC1R within the BNST is able to reduce dependence-induced excessive drinking as well as heightened anxiety-like behavior in rats exposed to alcohol vapors (Ferragud et al., 2021); hence, also considering the immunohistochemical data showing increased PACAP levels in the BNST in both models, we can conclude that chronic, intermittent exposure to ethanol consistently recruits the PACAP system of the BNST. Therefore, although chemogenetic approaches manipulate entire neuronal populations rather than individual neurotransmitter systems, we hypothesize that the increased PACAP release onto BNST neurons acts via PAC1R to mediate heavy alcohol drinking.

These data strongly suggest that the PACAP population mediating alcohol-related behaviors and alcohol drinking originates in brain regions sending PACAP projections to the BNST, consistent with the lack of reported PACAP mRNA in the mouse BNST. The type of approach we used here does not differentiate among specific inputs to the BNST, and, therefore, experiments directly manipulating a single PACAPergic pathway to the BNST will be necessary to ascertain the exact origin. Although multiple brain regions that project to the BNST are known to contain PACAP neurons (e.g., paraventricular nucleus of the hypothalamus, paraventricular nucleus of the thalamus, medial prefrontal cortex, dorsal vagal complex, and LPB; Weller and Smith, 1982; Légrádi et al., 1994; Kozicz et al., 1997; Missig et al., 2014; Missig et al., 2017; Kirry et al., 2018), recent studies have suggested that the majority of PACAP terminals found in both the BNST and the CeA originates in the LPB, which is recognized as a critical source of the peptide in these regions (Missig et al., 2014, 2017; Zhang et al., 2021; Jiang et al., 2023). In line with these observations, we confirmed the presence of mCherry in the LPB of the mice injected with the retrograde AAV in the BNST. In addition, we verified that our retrograde AAV only infected PACAP-expressing neurons and not another neuronal population also present in the LPB, Tac1-expressing neurons. The LPB, and in particular the external subnucleus, is indeed densely populated with PACAP-positive neurons that coexpress the glutamatergic marker VGluT2 and that largely overlap with CGRP-expressing neurons; these PACAP neurons mainly innervate the extended amygdala (Missig et al., 2014, 2017; Zhang et al., 2021; Pauli et al., 2022; Jiang et al., 2023). Interestingly, the chemogenetic activation of either the LPB-BNST or the LPB-CeA PACAP projection enhances anxiety-like behavior (Boucher et al., 2022; Seiglie et al., 2023), while the effects of the direct stimulation of these pathways on alcohol-related behaviors has not yet been investigated. Since axonal transport does not allow the visualization of clear peptide immunostaining in cell bodies, future studies will assess the effects of alcohol drinking on PACAP and CGRP mRNA expression in LPB, also assessing their coexpression.

Our experiments included both male and female mice. The BNST, in general, has a plethora of sexually dimorphic behavioral effects (Lebow and Chen, 2016). In the context of the PACAP/PAC1R system, sex differences have been reported in the context of stress-related behaviors and of nicotine effects (Ressler et al., 2011; King et al., 2017; Nega et al., 2020; Clancy et al., 2023; Slabe et al., 2023), and basal levels of PACAP were shown to be higher in specific brain regions of female animals (Curtis et al., 2023). On the other hand, other studies have reported no sex differences in the role of the PACAP/PAC1R system (Rajbhandari et al., 2021). In this study, we observed higher levels of PACAP in the BNST in female mice, which strengthens the notion of a sexual dimorphism of the PACAP system also in this brain area and perhaps suggests that higher endogenous levels of PACAP may predispose females to drink more alcohol. However, the effect of chronic alcohol drinking on PACAP levels did not differ in the two sexes. Similarly, no sex differences were observed in the effects of alcohol on CGRP and PAC1R levels, and no sex differences were found in alcohol drinking following the manipulation of the PACAP pathways to the BNST, suggesting a lack of sex differences in the action of chronic alcohol on these pathways. Future studies may address the role of sex hormones in the role of PACAP in alcohol-drinking behavior.

This study has a few limitations. The effects of the inhibition of PACAP afferents to the BNST were not assessed on the intake of an alternative reinforcer, as for example sucrose, which is useful to determine the selectivity of the effects for alcohol. In addition, the chemogenetic study did not include a control, non-DREADD virus to rule out a potential intrinsic effect of the DREADD ligand CNO on alcohol drinking; however, several studies have now shown that these doses of CNO do not affect ethanol intake per se in rodents (den Hartog et al., 2016; Kreifeldt et al., 2022; Suresh Nair et al., 2022; Griffin et al., 2023; Khan et al., 2023; Zamudio et al., 2023).

Together, these data suggest that BNST PACAP, possibly originating from cell bodies in the LPB, has a central role in driving heavy alcohol drinking in mice. This system may, therefore, represent a promising target for the development of novel treatments for AUD.

## Synthesis

Reviewing Editor: Leandro Vendruscolo, National Institute on Drug Abuse Decisions are customarily a result of the Reviewing Editor and the peer reviewers coming together and discussing their recommendations until a consensus is reached. When revisions are invited, a fact-based synthesis statement explaining their decision and outlining what is needed to prepare a revision will be listed below. The following reviewer(s) agreed to reveal their identity: Brendan Tunstall, Marsida Kallupi.

N/A
