# Supplementary figures and images for: Pituitary Adenylate Cyclase-Activating Polypeptide (PACAP) of the Bed Nucleus of the Stria Terminalis Mediates Heavy Alcohol Drinking in Mice
Source: eNeuro. 2023 Dec 15;10(12):ENEURO.0424-23.2023. doi: 10.1523/ENEURO.0424-23.2023 (PMC10755645; doi:10.1523/ENEURO.0424-23.2023)

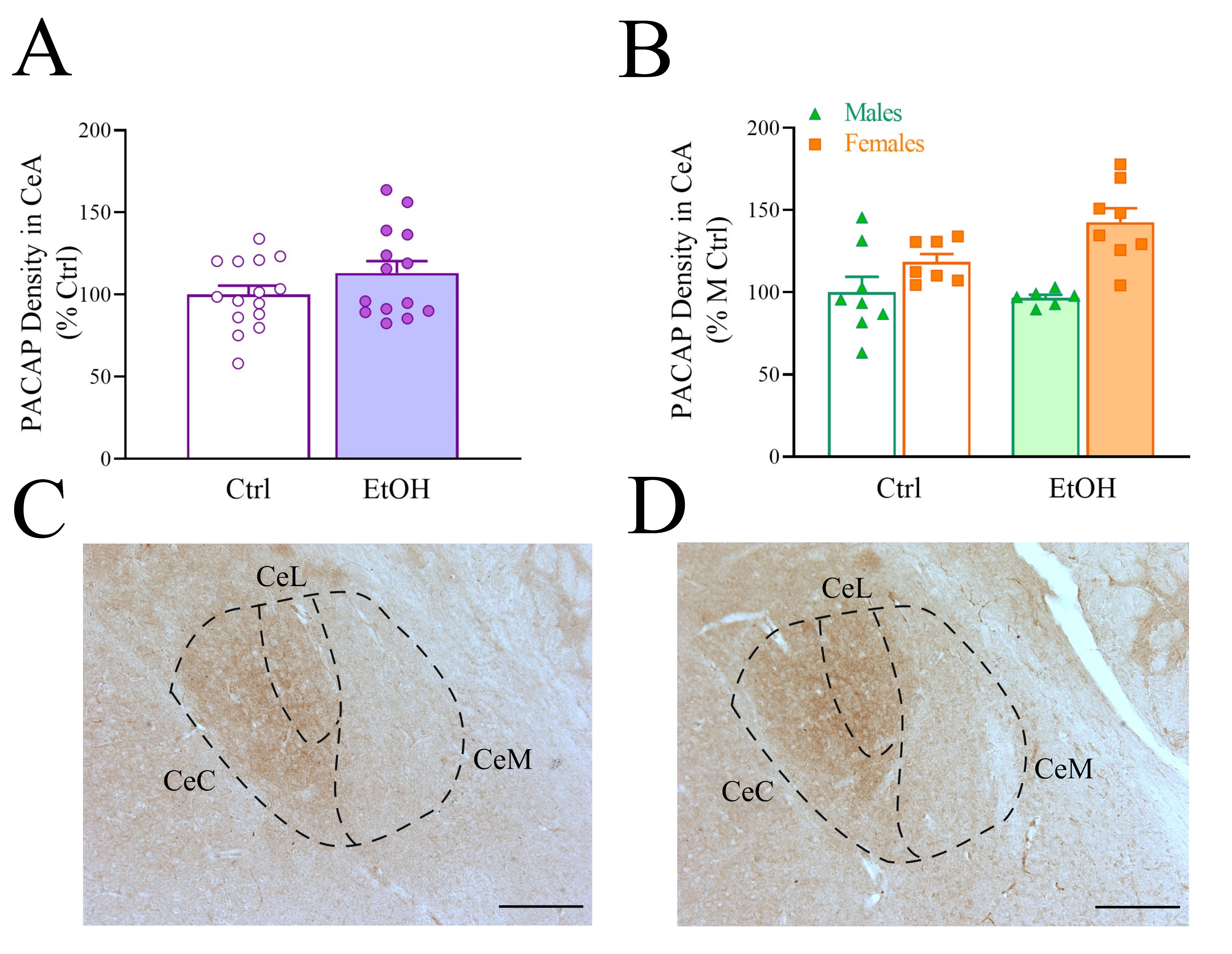

Supplement: Figure 2-1 — A, PACAP levels are unaltered in the CeA of male and female C57BL/6J mice exposed to intermittent access to alcohol (IA2BC; EtOH), compared with control, water-only mice (Ctrl). B, Data are reported with the two sexes pooled. C, D, Representative PACAP staining (DAB) in mouse BNST of a Ctrl and an EtOH subject, respectively. Magnification, 10×. Scale bar, 200 μm. Mice were killed after 7 weeks of IA2BC, 24 h after the end of the last drinking session. CeM, CeA medial. Data represent the mean ± SEM (A, n = 14–15/group; B, n = 6–8/group). Download Figure 2-1, TIF file. [file enu-eN-NWR-0424-23-s02.tif]

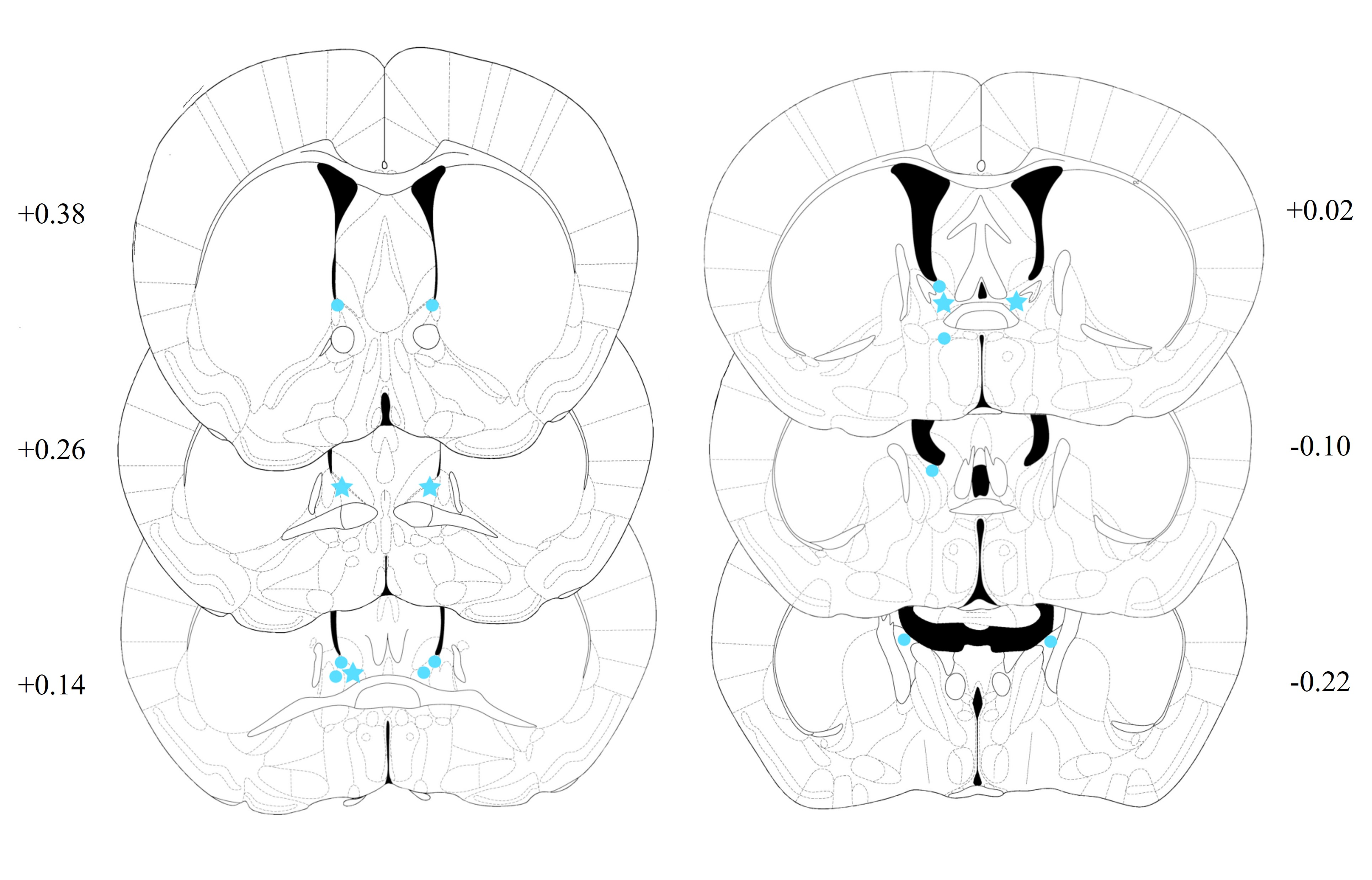

Supplement: Figure 4-1 — A, Illustrations of coronal rat brain slices; numbers represent the distance from bregma (mm). Symbols represent the injection sites of the AAVrg-hSyn-DIO-hM4D-mCherry in the BNST (each dot is the location of one injection, each star the location of 2 or more injections). Download Figure 4-1, TIF file. [file enu-eN-NWR-0424-23-s03.tif]
